# Tracheal Stenosis and Congenital Heart Disease in Trisomy 21

**DOI:** 10.3390/children6090098

**Published:** 2019-09-04

**Authors:** Ranjit I Kylat

**Affiliations:** Department of Pediatrics, University of Arizona, College of Medicine, Tucson, AZ 85724, USA; rkylat@gmail.com; Tel.: +1-520-626-6627; Fax: +1-520-626-5009

**Keywords:** tracheal stenosis, tracheal rings, complete, trisomy 21, congenital heart disease, bronchoscopy, slide tracheoplasty

## Abstract

Tracheal rings (TR) are rare, congenital cartilaginous defect of the upper airway and are usually due to complete or near complete circumferential cartilaginous tracheal rings, with variable degrees of tracheal stenosis (TS) and shortening. Chromosomal anomalies like trisomy 21 are characteristically associated with a wide range of upper airway anomalies including TS and congenital heart disease (CHD). However, the overall prevalence of severe forms of TS is rare and reported in 1.2% of all CHD patients. Herein, we present a rare association of severe TS due to complete tracheal rings in a trisomy 21 patient with CHD and the challenges in the management.

## 1. Introduction

Tracheal rings (TR) are rare, congenital cartilaginous defect of the upper airway and are usually due to complete or near complete circumferential cartilaginous tracheal rings, with variable degrees of tracheal stenosis (TS) and shortening [1]. In the normal trachea, the cartilaginous rings are C-shaped but if the tracheal rings grow out of proportion to the posterior membranous trachea, there is restricted tracheal growth leading to TS [1,2]. In the more severe forms of TS, as in cases of complete tracheal rings (CTR), absence of tracheal pars membranacea creates an hourglass trachea [3]. Chromosomal anomalies like trisomy 21 are characteristically associated with a wide range of upper and lower airway anomalies due to the midfacial hypoplasia, larygomalacia, tracheomalacia, and tracheo-bronchial anomalies. Incidentally, congenital heart disease (CHD) occurs in about 40% of patients with trisomy 21, but the overall prevalence of the more severe forms of TS is rare and reported in 1.2% of all CHD patients [4]. Many of these patients require major cardiac repair and as it has implications in the preoperative, and postoperative management, a thorough assessment of the airway is needed. Herein, we present a rare association of severe TS due to CTR in a trisomy 21 patient with CHD and the challenges in the management.

## 2. Case

**Figure 1:**
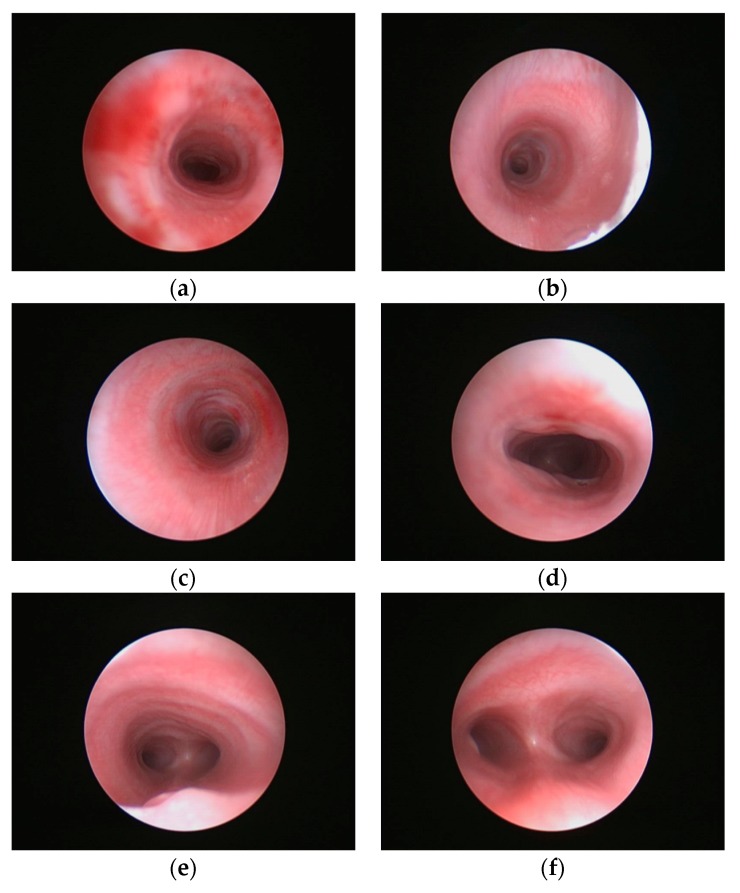
Direct bronchoscopic images: (**a**) to (**f**) – multiple bronchoscopic images revealing traumatic injury in (**a**) and multiple tracheal rings best seen in (**b**), (**c**) & (**e**).

A 38-year-old female, with a history of smoking, illegal drug use and poor prenatal care was diagnosed with fetal cardiac anomalies. She did not have any further prenatal testing. She delivered a male infant at term, who had respiratory distress at birth. As there was difficulty with intubation, bronchoscopy performed soon afterwards showed multiple CTR (Figure 1). Two days later he was extubated with stridor and mild retractions. Physical examination findings of trisomy 21 were confirmed on chromosomal analysis. Echocardiography revealed a partial atrioventricular septal defect. The patient was managed in hospital for three weeks and then underwent slide tracheoplasty and cardiac repair at 4 months age. At the last follow up at one year of age, he appeared well from a cardiorespiratory status with appropriate growth. 

## 3. Discussion

Congenital TS, although rare, affects 1 in 65,000 live births [2]. In 50%–75% of these cases it is associated with a cardiovascular anomaly, the most common of which is an aberrant left pulmonary artery which arises from the proximal part of the right pulmonary artery and loops behind the trachea but in front of the esophagus and causes the tracheal compression (pulmonary artery sling) [2,5,6]. TS is classified in the neonate as a long segment if it is more than 1 cm in length or involving more than 50% of the trachea if the diagnosis is not apparent prenatally [2]. In children with trisomy 21, the respiratory symptoms like stridor are common due to mid-facial and airway anatomical differences. The most common findings noted on endoscopy are laryngomalacia (50%), tracheomalacia (35%), and bronchomalacia (25%) [7]. However, routine laryngoscopy or bronchoscopy is not performed in all patients with respiratory symptomatology, and hence, there could be a higher incidence than reported. Vascular malformation such as left aortic arch with an aberrant origin of the right subclavian artery are also common in this population. In retrospective reviews, tracheal stenosis caused by complete tracheal rings was often identified in children with Down syndrome, and the clinical presentation and airway anatomy was noted to be extremely variable [8,9].

Resection and primary end-to-end anastomosis are possible in TS involving a very short segment. Anterior augmentation methods and rib cartilage, pericardial, and esophageal patch tracheoplasty have given way to slide tracheoplasty, which was first performed in 1989 [10]. This reconstruction of the stenotic trachea is done using native tracheal walls with its preserved blood supply. This is done by transecting at the midpoint of the stenosis, and posterior longitudinal incisions are made to widen the trachea before the anastomosis is performed [2]. Newer techniques using 3 D printed and stem cell-based tracheal transplants are being attempted [11,12].

In those undergoing cardiac surgery, it is imperative that an accurate respiratory diagnosis be made if there is persistent symptomatology, as it could affect the planning and risk stratification for surgery. The recommendation is for endoscopic direct visualization, but as part of the evaluation of cardiac anatomy, it may be possible to use three-dimensional turbo field echo magnetic resonance imaging for preoperative airway evaluation to demonstrate the tracheobronchial tree [13]. Its advantage is that it is not invasive and does not involve ionizing radiation [13]. In planning for surgery, it is ideal if cardiac surgery can be performed at the same time as tracheal repair, but in certain cases, an endoscopic bronchial stent may need to be placed as a temporizing measure in those patients with persistent symptoms [14,15]. In certain cases, the diagnosis could be missed for years as initial airway symptoms could be mild but could result in acute decompensation as exemplified in a previously reported case [16].

The key learning point to be highlighted is that in trisomy 21, both airway and cardiac anomalies coexist, and it would be prudent to thoroughly evaluate the airway in those patients with persistent symptoms or in those with CHD, needing interventions.
